# Recall of age of weaning and other breastfeeding variables

**DOI:** 10.1186/1746-4358-1-4

**Published:** 2006-03-09

**Authors:** Brenda Gillespie, Hannah d'Arcy, Kendra Schwartz, Janet Kay Bobo, Betsy Foxman

**Affiliations:** 1 Center for Statistical Consultation and Research, University of Michigan, Ann Arbor, MI 48109-1070, USA; 2 Department of Family Medicine, Wayne State University, 4201 St. Antoine, UHC-4J, Detroit, MI 48210, USA; 3 Department of Preventive and Societal Medicine, University of Nebraska Medical Center, Omaha, Nebraska 68198-4350, USA; 4 Department of Epidemiology, University of Michigan School of Public Health, Ann Arbor, MI 48109-2929, USA

## Abstract

**Background:**

Many studies of the impact of breastfeeding on child or maternal health have relied on data reported retrospectively. The goal of this study was to assess recall accuracy among breastfeeding mothers of retrospectively collected data on age of weaning, reasons for cessation, breast pain, lactation mastitis, and pumping.

**Methods:**

Women in Michigan and Nebraska, U.S.A. were interviewed by telephone every 3 weeks during the first 3 months after the birth of their child, and mailed a questionnaire at 6 months. A subset was interviewed again by telephone approximately 1–3.5 years after the birth. The results for the three recall periods, collected 1994–1998, were compared using correlation, linear and Cox regression analysis, and sensitivity and specificity estimates.

**Results:**

The 184 participants were aged 18–42, mostly white (95%) and 63% had an older child. The age of weaning tended to be overestimated in interviews 1–3.5 years after birth compared to those within 3 weeks of the event, by approximately one month for 1–3.5 year recall and two weeks for 6-month recall (p < 0.001 in both cases). Recall accuracy of reasons for weaning varied greatly by reason, with mastitis and return to work having the most recall validity. The sensitivity of 1–3.5 year recall of mastitis was 80%, but was only 54% for nipple cracks or sores.

**Conclusion:**

Breastfeeding duration among short-term breastfeeders tended to be somewhat overestimated when measured at 1–3.5 years post-partum. Reporting of other breastfeeding characteristics had variable reliability. Studies employing retrospective breastfeeding data should consider the possibility of such errors.

## Background

Many studies of the impact of breastfeeding on child or maternal health have relied on data reported retrospectively. For example, the U.S. National Maternal and Infant Health Survey (NMIHS) asked mothers to recall how many times a day they fed breast milk, formula, and other foods each month during the first 6 months of the baby's life[1]. Questionnaires were returned between 6 and 31 months after the birth, with a mean time since birth of 17 months. The U.S. Department of Health and Human Service's Healthy People 2010[2] uses the Ross Laboratories Mothers' Survey, a national survey to identify baseline rates of breastfeeding and monitor progress in meeting the national objectives. Previously mailed to new mothers at 6 months post-partum, it now collects information every month for the first 12 months post-partum. The 1981 National Health Interview Survey, conducted by the U.S. National Center for Health Statistics, included a Child Health Supplement that asked about childhood diseases for a randomly selected child in the household under 18 years of age, as well as about breastfeeding and supplementation practices and weaning age[3]. For this sample, the recall period for breastfeeding questions was up to 18 years. In case-control studies of the effects of breastfeeding on adult-onset diseases, questions about breastfeeding may be asked decades later. While prospective designs are preferable, they may not be feasible in some settings. It is therefore of interest to investigate the effect of recall bias on reported breastfeeding practices.

Recall bias is common among subjects interviewed about past events [4]. One might hypothesize that for breastfeeding, which often occurs at a time of stress and sleep deprivation, recall of past events might be especially prone to bias and/or imprecision. In addition, particularly in the U.S., social pressures to breastfeed might result in overestimates of breastfeeding time. We report here the results of a questionnaire about breastfeeding habits given every 3 weeks up to 12 weeks post-partum to breastfeeding women in the U.S., and given again at six months and at 1–3.5 years after the birth. Topics included the baby's age at weaning, reasons for cessation, breast pain, lactation mastitis, and pumping.

## Methods

This study was part of a larger study of breastfeeding habits of women[5], in which 946 women intending to breastfeed were enrolled before delivery, and those who breastfed for at least one day were followed until weaning or for up to three months after delivery. The research was approved by the institutional review boards at both the University of Michigan and University of Nebraska. Recruitment and interviewing were conducted between 1994 and 1998. These women, 235 from Omaha, Nebraska and 711 from the Detroit, Michigan metropolitan area were telephoned every three weeks for the first 12 weeks after the birth of their child and asked about breastfeeding and pumping habits, weaning, and incidence of breast pain or lactation mastitis. A questionnaire was mailed to obtain 6-month follow-up data.

Because of an error in the computerized skip pattern of the telephone interview, certain questions were inadvertently omitted from some of the interviews. In particular, women who had stopped breastfeeding in the previous three weeks and had never expressed milk skipped all questions regarding mastitis and breastfeeding habits for that interview. When the skip problem was discovered after recruitment was complete, the women with the "bad skip" (n = 54) were called back to recover the answers to the missed questions. Because participants had entered the study over approximately a 3-year interval, the recall period was between approximately one and 3.5 years. We were concerned about the quality of recall. Several women had delivered another child during the interim period, and were breastfeeding the younger child at the time of the telephone call. Confusion and reporting errors could easily occur in this context. To address these concerns, we conducted a validation study on a subset of women from the larger study of breastfeeding and mastitis.

Because the original study question related to lactation mastitis, we called all 74 women who had originally reported having mastitis to check for recall bias among these women for whom we had the "true" response. We also called 96 randomly selected women whom we had originally interviewed but who did not have the "bad skip", and who had not reported lactation mastitis. We were able to re-interview 74 (77%) of the 96 randomly selected women, 66 (89%) of the 74 women who had reported mastitis, and 44 (81%) of the 54 women with "bad skips", for a total of 184 women (Table 1). For items not involved in the "bad skip" (e.g., age of weaning, reasons for cessation), all 184 women were included in the analyses. For items involved in the bad skip (e.g., nipple cracks or sores and mastitis), those 44 women involved in the bad skip were handled separately as noted in the tables. We refer to the child who was being breastfed during the original interview as the "index child".

**Table 1:** Sub-samples of the 184 breastfeeding women used for each table and figure of the paper. All 184 women were telephoned for the original interviews every 3 weeks up to week 12, and were mailed a questionnaire at month 6. The 184 selected for the 1–3.5 year call-back included 66 with previously reported mastitis, 44 with an error in their original computerized questionnaire skip pattern, and 74 selected randomly.

	Total sample (n = 184)	Sub-samples of total (n = 184) sample		
		
		Weaned ≤ 3 months (n = 124)	With 6 month follow-up (n = 118)	Weaned ≤ 3 months and had 6 month follow-up (n = 95)
**Table 2**: Reasons for breastfeeding cessation		X		
**Table 3**: Breast pain, nipple cracks or sores, and mastitis	X^a^			
**Figure 1**: Life table estimates of the probability of still breastfeeding by age of infant			X	
**Figure 2**: Recalled length of breastfeeding plotted against initially measured length of breastfeeding for 6-month recall				X
**Figure 3**: Recalled length of breastfeeding plotted against initially measured length of breastfeeding for 1–3.5 year recall		X		

^a^Some exclusions apply, as described in Table 3 footnotes.

The initial interviews at 3, 6, 9, and 12 weeks post-partum asked the date of weaning the child ("When did you stop breastfeeding?" (mo/day/yr)), and accepted the week (1^st^, 2^nd^, or 3^rd ^of interval since last interview) if the respondent could not remember the exact day. We considered "stopping breastfeeding" to refer to cessation of breastfeeding the baby, which would not preclude continuing to pump milk for later bottle feeding. We did not make a distinction between exclusive breastfeeding and breastfeeding supplemented with other foods. The 6-month interview also asked the date of weaning ("When did you stop breastfeeding?" (mo/day/yr), with instructions, "If uncertain of exact day, give [calendar] month and year." The 1–3.5 year interviews asked, "How many months old was [baby's name] when he/she stopped breastfeeding?" The age of weaning was asked in months because recall in weeks or days was not expected to be accurate. Breastfeeding duration was analyzed in months, with initially reported weeks grouped into months as follows: 1–4 weeks (less than 1 month); 5–8 weeks (1 to <2 months); and 9–12 weeks (2 to <3 months). If the weaning time at the 6-month interview was reported as an exact date, then recall could be evaluated without bias. If only the month and year were given, we imputed the weaning to have occurred on the 15^th ^of the month, which would overestimate approximately half by up to 15 days, and underestimate the other half by up to 15 days. For weaning times reported at 1–3.5 years, mothers may have reported the nearest whole month by rounding, or by giving the last whole month of age achieved (e.g., as we report age in years to be, say, 25, even if it is the day before the 26^th ^birthday). We assumed the latter convention when defining the groupings of weeks into month intervals listed above. If women used rounding to the nearest month rather than the "birthday" convention of reporting weaning month, they might over-report the weaning age. For example, a baby weaned at 7 weeks would be grouped into the 1-month weaning interval by initial report, but a "rounding" mother would report the age as 2 months. Thus, we expect some over-reporting of weaning age by up to one month due entirely to our method of data collection.

Statistical methods for comparing continuous response variables between pairs of interviews for the same woman included scatterplots, correlation, and regression. For categorical variables, McNemar's test and Cohen's kappa statistics were used to compare the initial (every 3 week) and longer-term recalled responses. In addition, sensitivity (proportion recalling the event among those who had reported it initially) and specificity (proportion not recalling the event among those not reporting it initially) were calculated, assuming that the initial responses reflected the truth. Among women weaning within the first three months, predictors of recall error in age of weaning were explored using multiple regression. Both the signed difference (recall minus initially-reported weaning month) and the corresponding absolute difference were analyzed. For absolute recall error (ignoring the sign of the difference), logarithms were taken to reduce skewness; for these models, covariate effects are presented in terms of percent differences. These analyses were performed using SAS [6] and SPSS [7] statistical software.

The distributions of age at weaning were estimated using life table methods [8], with censoring in each interval assumed to be at the end rather than the middle of the interval. Ages of weaning based on the initial, 6-month, and 1–3.5 year reports were compared using Cox regression, with adjustment for site (Omaha and Detroit) and with clustering by respondent to adjust for the correlation between reports for the same child [9]. Because the comparisons between recall periods are within woman, the statistical power to detect differences is higher than would be expected by comparing the life-table estimates for the individual recall periods. The survival analyses were performed using Splus [10] statistical software.

## Results

Of the 946 women who were interviewed every 3 weeks during the first three months after the birth of their child, 690 (73%) returned the 6-month questionnaire. In our validation study of the subset of women selected for call-back, 184 were interviewed by telephone approximately 1 to 3.5 years after the birth. Data for all three recall periods (initial, 6-month, and 1–3.5 year recall) were available for 118 (64%) women. Most analyses presented here compare the initial reports to the 1–3.5 year recall, based on the 184 women interviewed 1–3.5 years after the birth, but the results for time to weaning are presented for both the 6-month and 1–3.5 year recall periods (Table 1).

Of the184 women interviewed during the 1–3.5 year recall period, 83 were from Omaha and 101 were from Detroit. Women ranged in age from 18 to 42, with a mean age of 30. Respondents were 95% white, 3% black, and 2% other races, and 96% were married or living with a partner. Most respondents (63%) had a child older than the index child, and 33 women (18%) had delivered an infant after the index child. Annual family income was reported to be less than $25,000 for 12% of the women, and greater than $50,000 for 48% of the women. (For comparison, US median family income for a 4-person family in 1998 was $56,061.) The times between the birth of the index child and the call-back ranged from 9 to 43 months, with a mean of 25.5 months. We refer to this interval as 1–3.5 year recall, although 8 (4%) women were interviewed between 9 and 11 months, and 1 was interviewed at 43 months.

Women contacted at the 1–3.5 year call-back were compared with those who could not be contacted with respect to demographic and breastfeeding variables. No differences were found in mother's education level or parity, but those re-contacted were slightly older (30 vs. 28 years on average, p = 0.01) and tended to have higher incomes (48% vs. 30% above $50,000, p = 0.006). Women contacted were slightly less likely to have stopped breastfeeding during the first 3 months (67% vs. 82%, p = 0.08), as compared with women not contacted. Only 3 women who were contacted refused the re-interview.

### Recall of age of weaning

Based on the initial interviews, 124 (67%) of the 184 women stopped breastfeeding during the first three months. By the time of the call-back interview 1 to 3.5 years later, almost all women (95%) had ceased breastfeeding the index child. For the comparison of reported age of weaning among the initial interview, 6-month questionnaire, and 1–3.5 year interview, most analyses were based on the subset of 118 women who had data for all three recall periods.

**Figure 1:**
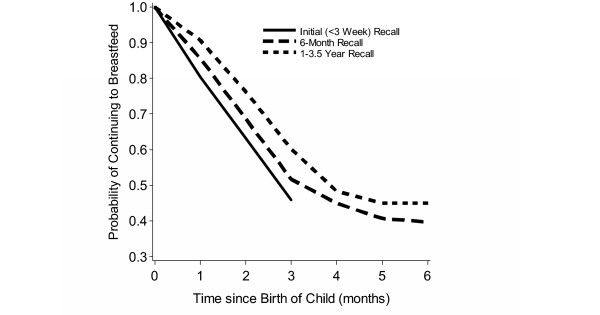
Life table estimates of the probability of continuing to breastfeed by age of infant, from initial (every 3 week) interviews (solid line), 6-month recall (dashed line), and 1–3.5 year recall (dotted line). Only women with data for all three recall periods were included (n = 118).

Life-table estimates of the probability of continuing to breastfeed by time since birth for the initial interview, the 6-month questionnaire, and the 1–3.5 year interview are given in Figure 1. The probability of continued breastfeeding at three months post-partum was estimated to be 0.46 (95% CI [0.35, 0.56]) based on the initial interview, 0.52 (95% CI [0.42, 0.61]) based on the 6-month questionnaire, and 0.60 (95% CI [0.51, 0.69]) based on the 1–3.5 year interview. Estimates of median breastfeeding duration increased with recall time: 2.8 months reported initially, 3.2 months with 6-month recall, and 3.9 months with 1–3.5 year recall, showing an average overestimation of approximately one month for 1–3.5 year recall.

Separate statistical comparisons of time to weaning were made for initial report versus 6-month recall, initial report versus 1–3.5 year recall, and 6-month versus 1–3.5 year recall. The analyses were adjusted for site because women in Omaha tended to wean much earlier than did the women in Detroit. Six-month recall times were significantly longer than initially-reported weaning times (HR = 0.82, 95% CI (0.73, 0.92), p = 0.0011, n = 118). A hazard ratio of 0.82 indicates that the rate (approximately, risk) of reporting having weaned by a given time is 18% lower based on 6-month recall compared to 3-week recall. One to 3.5 year recall times were also significantly longer than initially-reported weaning times, with a similar effect using all available data (HR = 0.75, 95% CI (0.71, 0.80), p < 0.0001, n = 184) or only cases with data in all three recall periods (HR = 0.78, 95% CI (0.72, 0.85), p < 0.0001, n = 118). Although the 1–3.5 year recall times were slightly longer than the 6-month recall times (HR = 0.95, 95% CI (0.83, 1.08), this difference was not significant (p = 0.43, n = 118). There was no significant difference in the recall effect between Detroit and Omaha.

**Figure 2:**
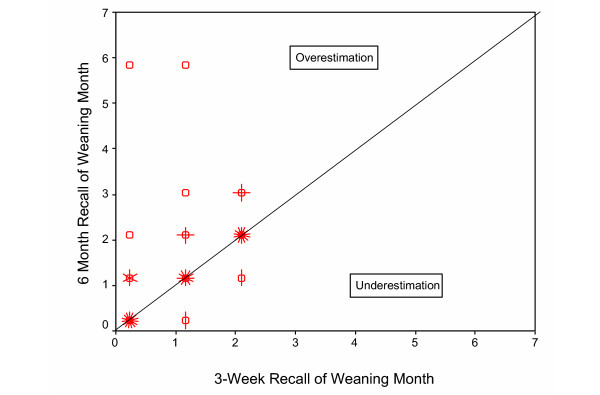
Recalled length of breastfeeding plotted against initially measured length of breastfeeding for 6-month recall (n = 95) for women who stopped breastfeeding during the first three months post-partum. Initial interviews were done at 3, 6, 9, and 12 weeks post-partum, and asked about breastfeeding during each of the three one-week periods. The number of "petals" at each point represents the number of observations (up to approximately 20), but a single observation at a point is represented by an open circle with no petals. The plotted month represents an interval starting at the given month and extending until just prior to the next month. The correlation coefficient is 0.49 (95% CI [0.32, 0.63]).

**Figure 3:**
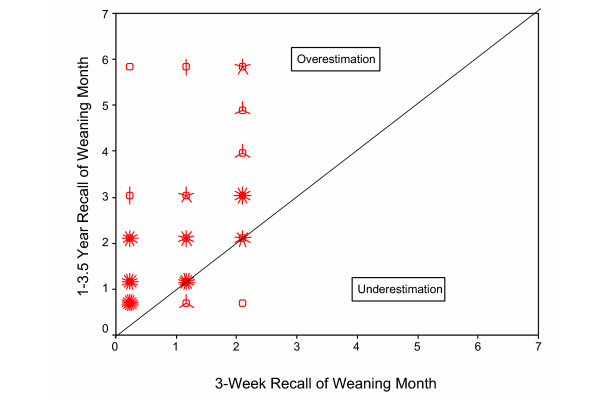
Recalled length of breastfeeding plotted against initially measured length of breastfeeding for 1–3.5 year recall (n = 124), for women who stopped breastfeeding during the first three months post-partum. Initial interviews were done at 3, 6, 9, and 12 weeks post-partum, and asked about breastfeeding during each of the three one-week periods. The number of "petals" at each point represents the number of observations (up to approximately 20), but a single observation at a point is represented by an open circle with no petals. The plotted month represents an interval starting at the given month and extending until just prior to the next month. The correlation coefficient is 0.59 (95% CI [0.46, 0.69]).

For women who initially reported weaning within 3 months, scatter plots of the times of weaning reported at 6 months (n = 95) and 1–3.5 years (n = 124) versus the times of weaning reported at the initial interviews are shown in Figures 2 and 3, respectively. The correlation between initial report and longer-term recall weaning times was only 0.49 (95% CI [0.32, 0.63]) for 6-month recall and 0.59 (95% CI [0.46, 0.69]) for 1–3.5 year recall. Both Figures 2 and 3 illustrate that it is more common to overestimate than underestimate, and also illustrate that the overestimation can be extreme in some cases. Discrepancies of one month in either direction could be attributed to rounding errors, but larger discrepancies are difficult to explain. Of the 58 women reporting still breastfeeding at the original 12-week interview, only 1 (2%) reported stopping before 3 months on re-interview. It is possible that some women weaned but continued pumping, and then resumed breastfeeding at a later time. Of the 61 women for whom we have a complete set of initial interviews and who provided the relevant information, 20% reported pumping after weaning. However, 27% (33/124) of women weaning within 3 months overestimated the weaning time by more than a month at 1–3.5 year recall. Thus, while this scenario may account for some of the overestimation, it does not appear to completely explain it.

Possible predictors of recall problems between initial and 1–3.5 year recall data were investigated among the 124 women who stopped breastfeeding during the first 3 months. Using regression analysis, we predicted the difference between ages given for weaning (longer-term recall minus initial 3-week recall) using the following variables: length of recall window, age at weaning (from initial data), study site, parity, mother's age, mother's education, family income, reason for stopping breastfeeding, previous breastfeeding experience, having had additional children in the interim, hours of sleep nightly at recall, and the baby's gender. Although no predictors were strongly significant, several were marginally significant, including length of recall (p = 0.09) and age of weaning (p = 0.05). Overestimation of length of breastfeeding was associated with both a longer recall interval (approximately one extra month for each additional 10 months of recall interval) and older age at weaning (those stopping in the first month tended not to overestimate, but those stopping in the third month tended to overestimate by a month on average). We also considered the absolute value of the difference between reported age at weaning, to study overall imprecision without focusing on over-estimation. Variables showing any association with less precise recall included greater parity (p = 0.10, with a 10% increase in error with each additional child), older age at weaning (p = 0.02, with a 7% increase in error for each additional week of breastfeeding, up to 12) and having previously breastfed a child (p = 0.08, with a 23% increase in error with previous breastfeeding). The proportion of variation explained by all significant variables combined (R^2^) was only 5% for the signed difference and 7% for the absolute difference.

### Recall of reasons for weaning

When weaning was reported in either the initial or 1–3.5 year interviews, the reasons for stopping breastfeeding were probed by asking specifically about seven possible reasons (listed in Table 2) as well as giving the opportunity to provide other reasons. The responses did not always agree between the initial and the 1–3.5 year interview among the 124 women who stopped breastfeeding during the first 3 months (Table 2). For example, "not enough milk" was given as a reason for cessation by 37 women at the original interview, and by 34 at re-interview. However, only 20 women reported that reason at both interviews. Of the 37 women giving this reason for cessation at the original interview, 17 (46%) failed to mention it on re-interview. Of the 87 women not mentioning it at the original interview, 14 (16%) mentioned it on re-interview. The kappa statistic was 0.389 (p < 0.001), indicating modest but significantly better than chance agreement between original and recall data. McNemar's test was not significant, indicating reasonably symmetric recall errors. Among the other reasons for breastfeeding cessation, the proportions of women reporting the reason at re-interview among those reporting it at original interview (i.e., sensitivity) ranged from 100% (mastitis) to 0% (baby stopped). On the other hand, the proportions of women not reporting the reason at re-interview among those not reporting it at original interview (i.e., specificity) ranged from 99% (mastitis, family objection) to 84% (not enough milk). Kappa statistics ranged from -0.07 (baby stopped) to 0.85 (mastitis), with the strongest associations for mastitis and return to work. McNemar test p-values ranged from 0.002 to 1.000, with significant lack of symmetry only observed for inconvenience. Recall accuracy of the reasons for cessation was quite heterogeneous, with mastitis and return to work having the most recall validity.

**Table 2:** Reasons for breastfeeding cessation reported 1–3.5 years post-partum (re-interview) compared to initial interviews within 3 weeks of breastfeeding cessation. Includes only women who had stopped breastfeeding within 3 months of the baby's birth (n = 124)

	**Reported During Initial Interviews (3, 6, 9, or 12 weeks post-partum)**							
	**Yes**		**No**					
					
	**Re-interview**		**Re-interview**					
**Reason^a^**	**Yes**	**No**	**Yes**	**No**	**Sensitivity^b^**	**Specificity^c^**	**Cohen's Kappa**	**McNemar's** **Test p-value**
**Not enough milk**	20	17	14	73	54%	84%	0.39**	0.720
**Back to work**	31	9	13	71	78%	85%	0.60**	0.523
**Inconvenient**	7	22	5	90	24%	95%	0.24**	0.002
**Painful**	8	14	8	94	36%	92%	0.32**	0.286
**Mastitis**	3	0	1	120	100%	99%	0.85**	1.000
**Family objected**	0	0	1	123	--	99%	--	--
**Baby stopped**	0	8	9	107	0%	92%	-0.07	1.000

^a ^Reasons are not mutually exclusive; women could report more than one reason.

^b ^Percentage of women reporting this reason at re-interview, out of those originally reporting it.

^c ^Percentage of women not reporting this reason at re-interview, out of those not originally reporting it.

* p < 0.05 ** p < 0.01

### Recall of breast pain, nipple cracks or sores, and mastitis in the first 3 months after birth

Breast or nipple pain was reported by 69% of women on initial interviews, and by 69% of women at the 1–3.5 year interview. However, only 50% of women reported pain at both interviews (Table 3). Of the 72 women reporting pain in both interviews, the reported week of first pain was quite different in the two reporting times (r = 0.15, 95% CI [-0.10, 0.38]). Cracks, fissures or sores were reported by 63% of women at original interview, and by 40% of women at re-interview, but only 35% reported these problems at both interviews. Of the women who reported these problems at both interviews, the reported weeks of first problem were only modestly correlated (r = 0.34, 95%CI [0.07, 0.57]). Recall of mastitis was fairly reliable among those initially reporting it (mastitis sensitivity 80%). Only two women who had not had mastitis claimed to have had it on re-interview (specificity 97%). Method of diagnosis (contact with health care provider in person or by phone) was reported fairly accurately. In-person diagnoses were claimed by 38% at initial interview and by 43% at re-interview, and 30% at both interviews. Reported week of first mastitis incidence was only weakly correlated between the interviews (r = 0.40, 95% CI [0.06, 0.65]), and number of occurrences of mastitis within the first 3 months (1, 2, or 3) were reported correctly on re-interview 76% of the time.

**Table 3:** Breast pain, nipple cracks or sores, and mastitis during the first 3 months after the baby's birth, and breast pumping before and after weaning: Initial interviews every 3 weeks until 12 weeks post-partum, and re-interview 1–3.5 years post-partum

	**Reported During Initial Interviews (3, 6, 9, or 12 weeks post-partum)**							
	**Yes**		**No**					
					
	**Re-interview**		**Re-interview**					
**Symptom/Illness**	**Yes**	**No**	**Yes**	**No**	**Sensitivity^a^**	**Specificity^b^**	**Cohen's Kappa**	**McNemar's Test p-value**
**Any breast pain^c^**	67	24	27	18	74%	40%	0.14	0.780
**Any cracks or sores^c^**	45	35	7	47	56%	87%	0.40**	< 0.001
**Mastitis^d^**	53	13	2	72	80%	97%	0.78**	0.007
**Mastitis Dx in person^d,e^**	16	4	7	26	80%	79%	0.57**	0.549
**Pumped before weaning^f^**	42	20	9	36	68%	80%	0.46**	0.061
**Pumped after weaning^g^**	6	6	13	36	50%	74%	0.19	0.167

^a ^Percentage of women reporting this symptom at re-interview, out of those originally reporting it.

^b ^Percentage of women not reporting this symptom at re-interview, out of those not originally reporting it.

* p < 0.05 ** p < 0.01

^c ^Women with the "bad skip" or otherwise incomplete prospective data who had not reported the condition in the existing interviews were excluded.

^d ^All women with the "bad skip" (n = 44) are excluded. None of those women had reported mastitis prior to the bad skip.

^e ^Includes only women who answered the question on mastitis at both initial interview and re-interview.

^f ^Women who stopped breastfeeding in the first week are excluded.

^g ^Women who stopped breastfeeding in the week of the interview were excluded because data after weaning were not available.

### Recall of breast pumping

Women were asked if they expressed or pumped milk both in the month before and the month after weaning. Pumping milk before weaning was reported by 58% of women at initial interview and by 48% at re-interview, but only by 39% at both interviews. More women forgot that they had pumped (32%) than erroneously "remembered" that they had pumped (20%).

Pumping milk after weaning was reported by 20% of women at initial interview, and by 31% at re-interview, but only by 10% at both interviews. As above, a greater proportion of women forgot that they had pumped (50%) than erroneously "remembered" that they had pumped (27%).

## Discussion

Recall of the details of breastfeeding can be quite inaccurate. On a population level, the effects of recall errors are diminished by combining errors of omission and commission (for dichotomous outcomes), or errors of overestimation and underestimation (for continuous outcomes). For example, breast or nipple pain was reported by 69% of women at both initial interviews and 1–3.5 year recall. Yet 37% of these women changed their answers between initial and recall interviews. For length of breastfeeding, we observed a statistically significant but modest overestimation for both 6-month and 1–3.5 year recall, with a magnitude of bias only approximately one month at 1–3.5 year recall. At the individual level, however, the discrepancies between initial and longer-term recall data become more apparent. For example, the correlation between weaning ages collected initially (every 3 week interviews during breastfeeding) and 1–3.5 years later was only 0.59. The use of retrospective breastfeeding data to investigate associations with other factors such as disease incidence may be problematic. Such analyses require data at the individual level to be correct, and recall errors will likely bias any association toward the null. Differences between initial and longer-term recall weaning data were only modestly predicted by variables such as length of recall period, age of weaning, parity of the mother, and previous breastfeeding experience.

Variation in estimates of duration of breastfeeding with time of recall have been noted in several other studies. Eaton-Evans and Dugdale[11] reported on 75 Australian children whose mothers were asked to recall duration of breastfeeding at 1 to 10 years post-partum. Compared with clinic records, 79% of mother's reports were within one month, and 21% were different by 2 to 3 months. This result is fairly similar to our estimate of 27% reporting more than 2 months different at 1–3.5 years. However, they found approximately equal numbers of mothers who over- and under-estimated duration of breastfeeding, where we found that most mothers over-estimated at longer-term recall. We attribute our preponderance of over-estimates primarily to regression to the mean, because we could only include women who initially reported weaning at 3 months or less. Had we been able to include longer-term breastfeeders, we expect that we would have seen under-estimation of breastfeeding on recall among the long-term breastfeeders. A study by Kark et al. [12] among 74 women in Jerusalem that compared medical records to reports up to 20 years post-partum also found similar numbers of women over- and under-reporting breastfeeding duration. However, the values over-reported tended to be larger than the values under-reported, and the reported durations had greater variability than those obtained from the medical records. A study among 318 Bedouin Arab women in Israel[13] found that reported breastfeeding duration increased by almost a month between reports at 6 months versus 18 months post-partum. Another study from Quebec, Canada of 39 breastfeeding women[14] found an increase in reported duration of breastfeeding by almost four months between data collected at regular intervals post-partum (monthly up to six months, and every three months thereafter) and again 8 or more years later. A more recent U.S. study[15] used initial breastfeeding information reported in a large study of menstrual cycle patterns in young women starting in 1934, with a follow-up questionnaire in 1990 asking, among other things, about breastfeeding duration. No overall recall bias in breastfeeding duration was reported, but considerable recall error was seen (s.d. = 2.7 months for difference between initial report and subsequent recall), with substantial over-reporting of duration among the short (1–2 month) breastfeeders, and under-reporting of duration among the long (8–12 month) breastfeeders. These observations are consistent with an explanation of regression to the mean, where with two values drawn from a bivariate distribution, the largest values on first draw are more likely to be followed by smaller values, and similarly the smallest values by larger values.

Some study designs have used two interviews given at relatively short intervals apart, but often several years post-partum. In a sample of 1199 Malaysian women interviewed twice, four months apart, Haaga [16] found a high correlation (r = 0.91) between duration of breastfeeding of the first child, with ages from birth ranging from one to over 25. In a sample of 15 Australian women with children one to three years old, Arbon and Byrne [17] found high correlation (r = 0.99) between weaning ages reported two weeks apart in a test-retest format. Promislow [15] found a much lower correlation (r = 0.55) between weaning ages reported approximately 50 years apart. We observed similarly low correlations between reports of breastfeeding duration: r = 0.49 for 3-week recall versus 6-month recall and r = 0.59 for 3-week recall versus 1–3.5 year recall. However, our lower correlations were at least partially due to the shorter range of durations (limited to 12 weeks) for the 3-week recall interviews.

The tendency among short-term breastfeeders to overestimate length of breastfeeding may be entirely due to regression to the mean, although there may be a contribution due to the current social pressure in the U.S. to breastfeed. For example, the American Academy of Pediatrics (AAP) recommends breastfeeding for at least one year [18]. The web site of LaLeche League, a U.S. breastfeeding support group, advocates breastfeeding for "as long as the mother and the baby wish to breastfeed"[19], but cites the AAP recommendation and adds that "Many of the health advantages of breastmilk are dose related: the longer the baby receives breastmilk, the greater are the benefits." The Healthy People 2000 guidelines, developed at the U.S. National Institutes of Health, targeted an increase in breastfeeding. Articles in health magazines and newsletters also commonly recommend breastfeeding to enhance the baby's health. If a reason for the over-reporting of the age at weaning is related to social pressure to breastfeed for longer periods, then any bias observed in this sample may not apply to other populations where social pressures are different. While the observed effect may be applicable to primarily white, middle-class populations across the U.S. (such as our populations in both Detroit and Omaha), completely different biases may operate in other cultures.

Different definitions of breastfeeding duration have been used in other studies. For example, Bland et al. [20] considered exclusive breastfeeding (EBF), with the duration of EBF considered as the time from birth until the introduction of other foods, including water and infant formula. They found poor recall of EBF duration (79% sensitivity and 40% specificity at two weeks for 6–9 month recall versus 48 hour recall). Aarts et al. [21] further investigate trends over time in exclusive breastfeeding and complementary/replacement feeding, and provide useful guidance in questionnaire design to capture important breastfeeding information.

Reports of reasons for weaning such as "inconvenient", "painful", and "not enough milk" varied between 3-week and 1–3.5 year-recall. Women were more likely to initially report these reasons but omit them at the 1–3.5 year interview than to report them newly at that time. Reasons for weaning that were reliably reported on both initial and longer-term recall interviews were "return to work" and "breastfeeding mastitis", although the numbers with mastitis are small. Retrospective reporting of the occurrence of breast pain, nipple cracks or sores, and mastitis was only moderately accurate, and reporting of the timing of these events was quite inaccurate. Recall of pumping before and after weaning was also fairly inaccurate.

It appears that with the passing of time, the details of the breastfeeding experience fade, and only the most memorable events, such as return to work, are clearly retained. Our mean recall period was approximately 2 years. It appears that recall quality degrades quickly, so that within several months to a few years, many women forget the details of the breastfeeding experience. Even at 6 months, the ages of weaning reported by our participants were significantly longer than those reported initially.

One limitation of this study is the less than perfect re-contact rate. However, the contact rate was fairly high (83% overall), and we do not expect bias in this regard. Many young families move to larger homes as the family size grows, and we have no reason to believe that such moves (which made re-contact difficult) were related to breastfeeding factors. In addition, our investigation found few substantial differences between those contacted and not contacted in demographic and several breastfeeding factors. The 3 refusals after contact, while possibly reflecting an atypical group, should not have had much effect on the results. Another limitation is that this study was unable to address the question of any versus no breastfeeding. Some studies retrospectively ask whether the child was breastfed at all, and base analyses on this dichotomous variable. Unfortunately, we cannot estimate the proportion breastfeeding because all subjects in our study breastfed for at least one day. What is clear is that age at weaning is not accurately remembered even after relatively short periods (3 months). We found no similar literature for comparison. Nonetheless, these results suggest studies of breastfeeding should be conducted either prospectively or within a very short (<1 month) time window following weaning.

## Conclusion

Although prospective studies are preferred, it is not always feasible to perform such studies of breastfeeding practices. For example, in adult-onset diseases, a retrospective case-control design is most efficient to study the potential protective effects of breastfeeding, but necessarily depends on past recall of the mother's breastfeeding experience. National cross-sectional or longitudinal surveys can be an efficient method of collecting large amounts of data on a range of topics. However, these data almost always rely on respondent recall of one to several years. Based on our results, retrospectively-collected data on age at weaning and other breastfeeding variables should be used with caution.

## Competing interests

The author(s) declare that they have no competing interests.

## Authors' contributions

BWG directed the statistical analyses, and prepared the bulk of the manuscript. HD performed data management and statistical analyses and critiqued the manuscript. KS and JKB participated in the development of the study and critiqued the manuscript. BF, as principal investigator, conceived of and directed the study, and made a major contribution to drafting and editing the manuscript. All authors approved the final manuscript.
